# Nanoscale dihydroartemisinin@zeolitic imidazolate frameworks for enhanced antigiardial activity and mechanism analysis

**DOI:** 10.3389/fvets.2024.1364287

**Published:** 2024-05-01

**Authors:** Xiaoming Jiang, Yawei Li, Shuainan Liu, Hongyu Sun, Meiyu Zheng, Xi Wan, Wenhe Zhu, Xianmin Feng

**Affiliations:** ^1^College of Medicine, Yanbian University, Yanji, Jilin, China; ^2^School of Basic Medicine Sciences, Jilin Medical University, Jilin, Jilin, China

**Keywords:** dihydroartemisinin, *Giardia lamblia*, Zif-8, RNA-seq, metabolism

## Abstract

An artificial semisynthetic material can be derived from artemisinin (ART) called dihydroartemisinin (DHA). Although DHA has enhanced antigiardial potential, its clinical application is limited because of its poor selectivity and low solubility. The drug’s absorption has a direct impact on the cell, and mechanism research is limited to its destruction of the cytoskeleton. In this study, we used the zeolitic imidazolate framework-8 and loaded it with DHA (DHA@Zif-8) to improve its antigiardial potential. DHA@Zif-8 can enhance cellular uptake, increase antigiardial proliferation and encystation, and expand the endoplasmic reticulum compared with the DHA-treated group. We used RNA sequencing (RNA-seq) to investigate the antigiardial mechanism. We found that 126 genes were downregulated and 123 genes were upregulated. According to the KEGG and GO pathway analysis, the metabolic functions in *G. lamblia* are affected by DHA@Zif-8 NPs. We used real-time quantitative reverse transcription polymerase chain reaction to verify our results using the RNA-seq data. DHA@Zif-8 NPs significantly enhanced the eradication of the parasite from the stool *in vivo*. In addition, the intestinal mucosal injury caused by *G. lamblia* trophozoites markedly improved in the intestine. This research provided the potential of utilizing DHA@Zif-8 to develop an antiprotozoan drug for clinical applications.

## 1 Introduction

Giardiasis in the gastrointestinal tract of humans and other mammals can be caused by the parasitic protozoan *Giardia lamblia* (*G. lamblia*) (1). According to the World Health Organization (WHO), one of the most common intestinal parasites is *G. lamblia*, which is responsible for approximately 300 million infections (5–10% of the world population) (2, 3). The two life stages of *G. lamblia* are trophozoite, which cause giardiasis and cyst, which is infective. This later stage causes acute or chronic diarrhea as well as malabsorption, abdominal pain, and weight loss (4). Comon clinical treatments for giardiasis include 5-nitroimidazole and benzimidazole derivatives, paromomycin, quinacrine, furazolidone, and nitazoxanide (5). Multidrug resistance to these drugs, however, has been reported in humans and has caused about 20% of treatment failures (6, 7). Therefore, a more effective drug to treat giardiasis is needed.

An antimalarial drug, artemisinin (ART), has been extracted from the Chinese plant *Artemisia annua* (8, 9). In addition to high safety and antimalarial efficacy, ART has successfully treated many other parasitic infections, such as protozoan parasites that infect humans. These parasites include *Trypanosoma*, *Leishmania*, *Naegleria fowleri*, *Acanthamoeba castellanii*, and *G. lamblia* (10). Reportedly, ART’s antiparasitic activity at a 50% concentration is effective against *Toxoplasma gondii* (11). ART is also effective against trypanosomes, inhibiting Ca^2+^ ATPase activity in the parasitic membrane (12). ART and its derivatives are noncytotoxic and inexpensive. They also can be used effectively as antiprotozoal drugs in combination with other antiprotozoal drugs. As a result, ART has created new avenues for a variety of combination therapies to treat parasitic protozoans. An artificial semisynthetic derivative called dihydroartemisinin (DHA) has been obtained after reducing ART (13). DHA not only has a hydroxyl group but also retains the biologically reactive endoperoxide which mediates the antimalarial activity. Therefore, it can be used to enhance the antimalarial effects of ART (14). DHA also has higher efficacy, wider distribution, easier absorption, and lower toxicity than ART, making it more advantageous. Other factors, however, restrict the use of DHA as an antiprotozoal drug, including short circulation half-life, high first-passage metabolism, poor water solubility, and nonspecific delivery *in vivo* (15, 16). To address these limitations, it may be feasible to combine DHA and nanocarriers.

Emerging nanocarriers have been developed for biomedical applications, such as metal–organic frameworks (MOFs), which are a class of inorganic–organic hybrid porous polymers (17). Zeolitic imidazolate framework-8 (Zif-8) is composed of Zn^2+^ and 2-methylimidazole. Among the MOFs, Zif-8 is commonly used in energy storage, sensing, catalysis, and gas separation thanks to its chemical and thermal stability as well as its surface area (18, 19). Zif-8 nanoparticles (Zif-8 NPs) have excellent internal biodegradability and thermal stability. In a previous study, Zif-8 MOF NP-encapsulated DHA has been shown to enhance cancer therapy.

In this study, we designed the Zif-8 NP-encapsulated DHA to improve the clinical application of DHA as well as its antiprotozoal effects. The antiprotozoal effects of the prepared DHA@Zif-8 NPs were better than the free DHA. In addition, we used RNA-seq analysis to assess how the gene expression in *G. lamblia*-treated DHA@Zif-8 NPs changed, and we examined the possible mechanisms. According to RNA-seq results, 219 genes are involved in DHA@Zif-8 NPs treated with *G. lamblia*. The KEGG and GO enrichment analysis indicated that DHA@Zif-8 NPs may affect the metabolic functions in *G. lamblia*. DHA@Zif-8 NPs showed that the number of fecal *G. lamblia* cysts and intestinal trophozoites was significantly decreased in DHA@Zif-8 NPs *in vivo* compared with the infected groups. This result may have been caused by the encapsulation mechanism of DHA by Zif-8 NPs. The antiprotozoal effect of DHA@Zif-8 NPs has not yet been clarified at the molecular level. This study provides a new perspective on the application of ART and its derivatives in clinic applications.

## 2 Results

### 2.1 Preparation of DHA@Zif-8 NPs

**Figure 1:**
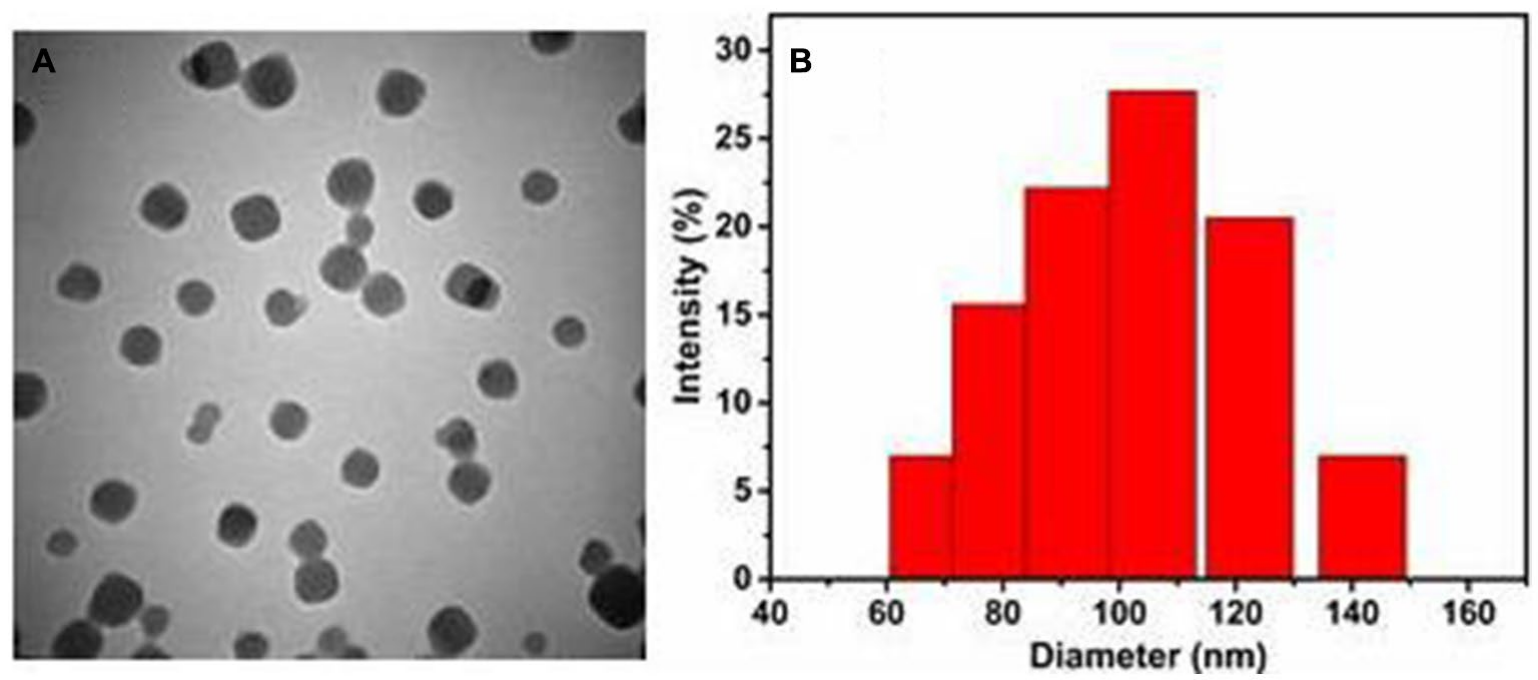
Basic characterization of prepared DHA@Zif-8 NPs. **(A)** A TEM image of DHA@Zif-8 NPs. **(B)** Particle size distributions of DHA@Zif-8 NPs dispersed in water.

We used an improved facile one-pot synthesis method to prepare DHA@Zif-8 NPs and pure Zif-8. We carefully screened and optimized the reaction conditions of reaction time, temperature, solvents, and the weight ratio of target drug molecules (DHA) to metal ions (Zn^2+^) and organic linkers (MIM). As the NPs formed, we loaded DHA into the framework of Zif-8 to circumvent the drawback of DHA’s poor water solubility. To investigate the sizes and morphologies of these NPs, we performed scanning electron microscopy (SEM) and transmission electron microscopy (TEM). The pure Zif-8 NPs and DHA@Zif-8 NPs both had uniform monodispersed particles and rhombic dodecahedral shapes. The average size of the particles was about 100 nm, as shown in Figure 1. We used the successfully prepared DHA@Zif-8 NPs as the basis to apply DHA clinically to treat *Giardia*.

### 2.2 Anti-*Giardia lamblia* effect of DHA@Zif-8 NPs *in vitro*

**Figure 2:**
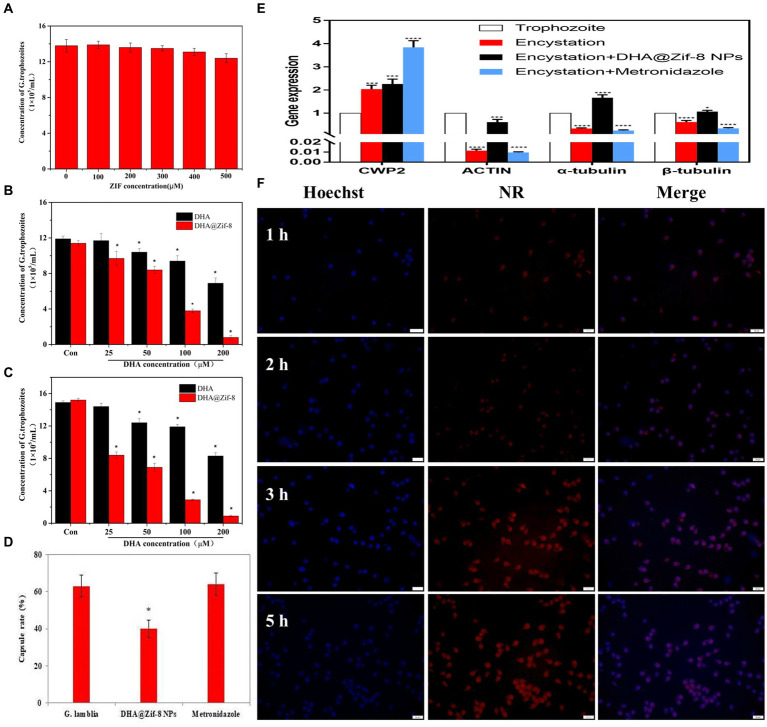
Effect of DHA@Zif-8 NPs on cell proliferation and cellular uptake of *G. lamblia*. **(A)** Cytocompatibility of Zif-8 NPs with *G. lamblia*. **(B,C)** The proliferation inhibition of *G. lamblia* after incubation with various levels of DHA@Zif-8 NPs or free DHA for 24 h or 48 h. **(D)** Effect of DHA@Zif-8 NPs on cysts formation. **(E)** Effect of DHA@Zif-8 NPs on cysts formation gene expression. **(F)** CLSM images of *G. lamblia* incubated with DHA@ZIF-8 NPs at 37°C for different times. Scale bars: 20 μm. Data are presented as mean ± SD, *n* = 5. *p < 0.05 compared with control.

To evaluate the *in vitro* antigiardial effects of DHA@Zif-8 NPs, we monitored the proliferation inhibition effect through cell counting. Because nanomaterials must be biocompatible for biomedical applications, we evaluated whether pure Zif-8 NPs demonstrated cellular toxicity on *G. lamblia*. As shown in Figure 2A, Zif-8 NPs exhibited negligible cytotoxicity toward *G. lamblia* at concentrations ranging from 0 to 500 μM, which indicated that Zif-8 NPs possess excellent biocompatibility. Compared with equivalent free DHA, the DHA@Zif-8 NPs had a much better proliferation inhibition effect on *G. lamblia*. The concentration range was between 0 and 200 μM for 24 or 48 h (Figures 2B,C). The IC_50_ value of free DHA on *G. lamblia* at 24 h was 233.3 μM and at 48 h was 221.7 μM. The DHA@Zif-8 NPs possessed IC_50_ values of 94.7 μM at 24 h and 31.1 μM at 48 h, which were significantly different from the free DHA.

During the encystation process of *Giardia* trophozoite, the changes in the expression of several genes are signs that cysts are forming. For example, the expression of α-tubulin, β-tubulin, and actin was decreased, and the expression of Cwp2 was increased. Metronidazole has been reported to promote the differentiation of *Giardia* trophozoites into cysts, which leads to resistance to metronidazole. Our results showed that the DHA@Zif-8 NP-treated group reduced cyst formation (Figure 2D). In the metronidazole-treatment group, the gene expression level of Cwp2 increased and the expression levels of actin, α-tubulin, and β-tubulin decreased. In the DHA@Zif-8 NP-treated group, the expression level of Cwp2 was increased as well as the expression levels of actin, α-tubulin, β-tubulin, and actin (Figure 2E). According to these results, *G. lamblia* cyst formation can be inhibited by DHA@ Zif-8 NPs.

The effective endocytosis of DHA@Zif-8 NPs may explain this enhanced toxicity. To improve drug effects, the endocytosis of NPs is essential. We used confocal laser scanning microscopy (CLSM) to detect the cellular uptake behavior of DHA@Zif-8 NPs by *G. lamblia*. We used a blue channel by Hoechst 33258 (a nucleus staining dye) to visualize the cell nuclei. To facilitate fluorescence observation, we embedded the DHA with Nile red (NR) into the Zif-8 NPs. After treatment of 50 μM of DHA@Zif-8 NPs, we observed a strong red fluorescence signal that was distributed mainly in the cytoplasm (Figure 2F). This result indicated that DHA@Zif-8 NPs passed into the cytoplasm from across the cell membrane. After a prolonged incubation time from 1 to 5 h, fluorescence intensity was significantly enhanced. This result showed that DHA@Zif-8 NPs possessed a time-dependent internalization, which validated the sustained uptake of the prepared DHA@Zif-8 NPs in successfully treating *G. lamblia*.

**Figure 3:**
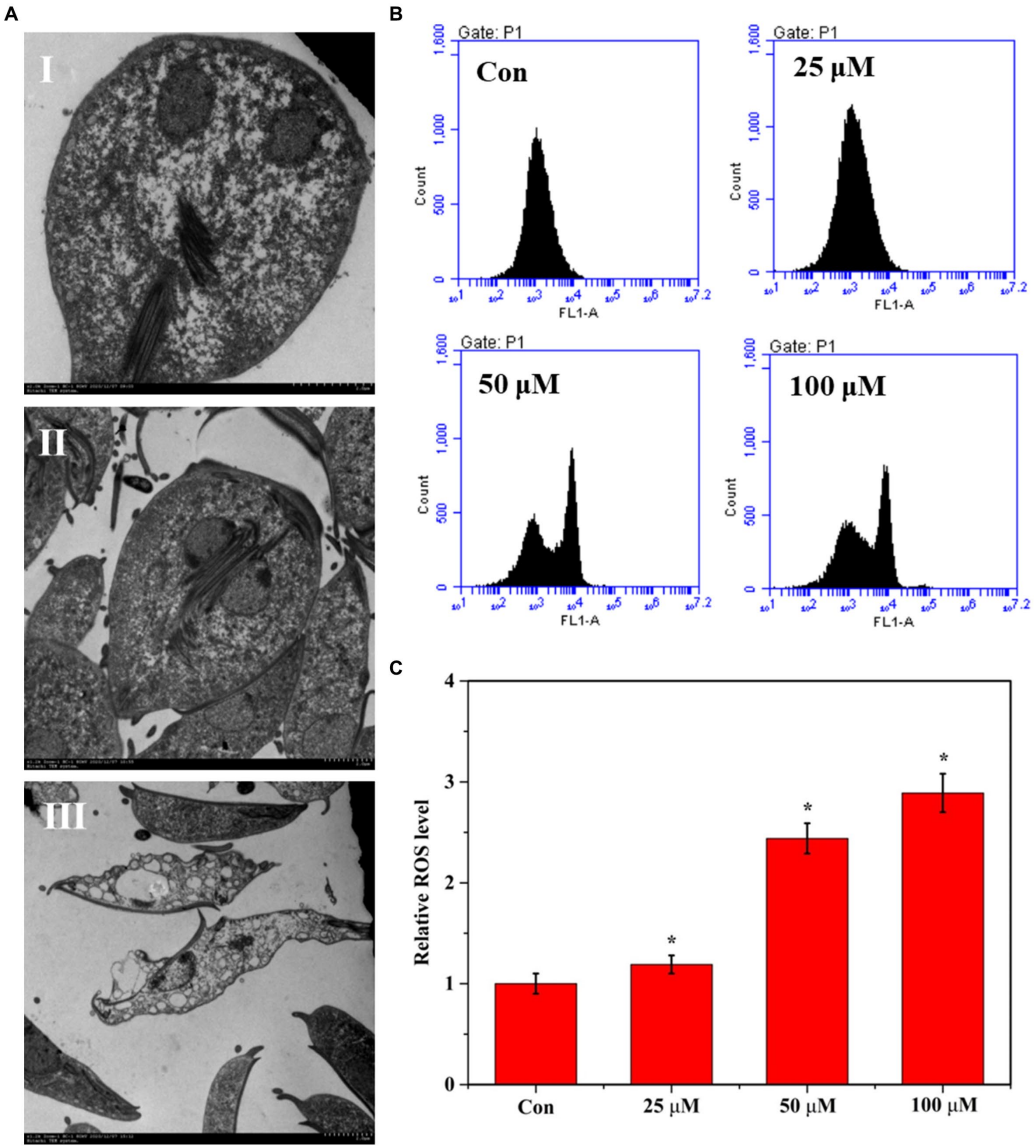
Effect of DHA@Zif-8 NPs on ultrastructural changes and ROS generation. **(A)** Ultrastructural changes of *G. lamblia* after treated with DHA@Zif-8 NPs. **(I)** Control group; **(II)** 25 μM DHA@ZIF-8 NPs; **(III)** 50 μM DHA@ZIF-8 NPs. **(B)** ROS generation in *G. lamblia*. **(C)** Quantitative analysis of ROS generation. Data are presented as mean ± SD, *n* = 5. *p < 0.05 compared with control.

We used SEM to observe the ultrastructural changes to explore the proliferation inhibition effects of Zif-8 NPs on *G. lamblia*. As shown in Figure 3AI, the cross-section of the Giardia trophozoite appeared pear-shaped, with two oval vesicular nuclei. The staining plasmids around the nucleus are evenly distributed at the inner edge of the nuclear membrane. Single-layer arranged small vesicles could be seen under the plasma membrane. Flagella are emitted from the matrix, and the sugar bodies in the cytoplasmic core are dense and evenly distributed. In the coronal section of the trophozoites, the cross-sectional view of the round nucleolus and flagella in the nucleus, the microtubules of the sucker around the axoneme of the tail flagella can be observed in the control group. After DHA@Zif-8 NPs treatment (Figures 3AII,III), the *G. lamblia* showed swelling expansion of the endoplasmic reticulum. Dissolution and vacuoles appeared in the cytoplasm. The microtubules of the sucker disintegrated. The cytoplasm is almost completely depleted, the nuclear chromatin edge gathers, perinuclear space widens, the plasma membrane breaks, and the ultrastructure of the nuclear membrane disintegrates.

### 2.3 Anti–*Giardia lamblia* mechanism of DHA@Zif-8 NPs

Reportedly, ART and its derivatives have demonstrated antiparasitic and antitumor activity by creating reactive oxygen species (ROS) in parasites and cancer cells. In this study, we performed flow cytometry to detect ROS in DHA@Zif-8 NP-treated *G. lamblia* to understand the potency of these treatments in generating ROS. Compared with the control group, the DHA@Zif-8 NP-treatment group generated more ROS. These results verified that DHA delivery by Zif-8 NPs to *G. lamblia* was improved and triggered significant inhibition *in vivo* following more ROS production (Figure 3B,C).

**Figure 4:**
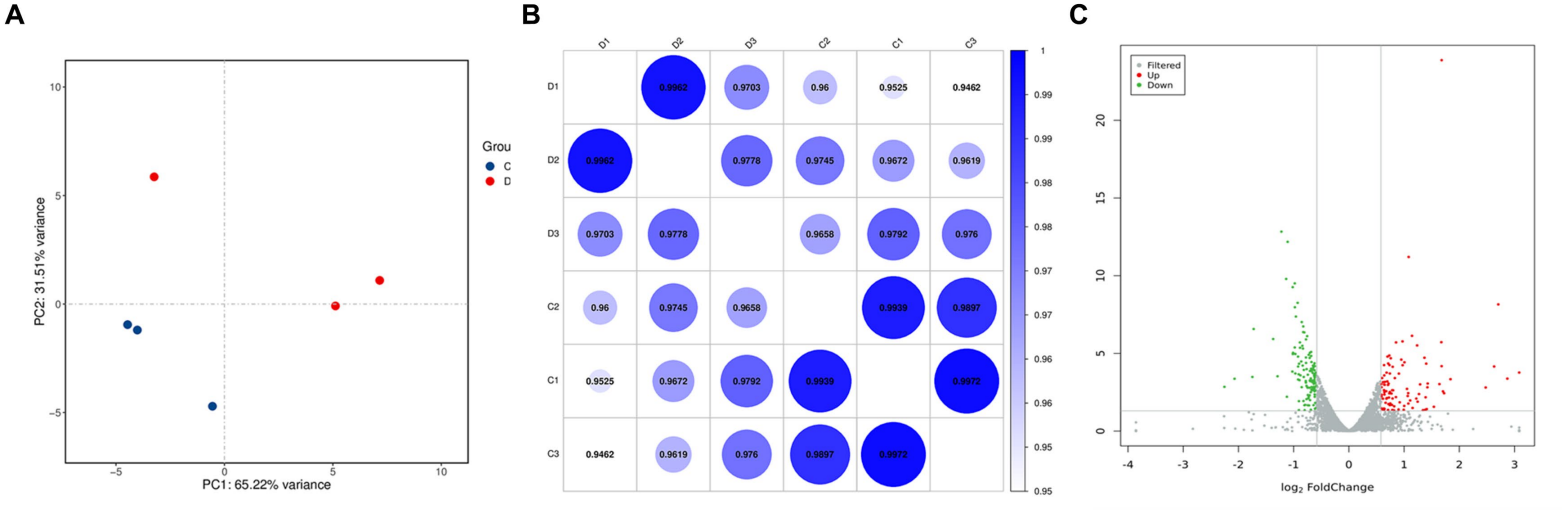
Principal component analysis (PCA) and volcano plots of differentially expressed genes (DEGs). **(A)** PCA analysis result. Each dot represents a sample. The blue represents C group and the red represents D group. **(B)** Correlation heat map demonstrating the association. **(C)** Volcano plots. Each dot represents a gene. The gray points represent a non-statistically significant difference in gene expression. The red field represents the up-regulated genes and the green field represents the down-regulated genes.

To verify the gene expression in *G. lamblia* before and after treatment with DHA@Zif-8 NP and to further explore the antigiardial mechanisms of DHA@Zif-8 NPs, we used RNA sequencing (RNA-seq). We used formaldehyde agarose gel electrophoresis and ultraviolet spectrophotometry to analyze the total RNA extracted from the DHA@Zif-8 NP-treated group (D) and untreated group (C) for integrity and quality. According to these results, the RNA obtained was nondegraded, complete, and suitable for RNA-seq analysis. We used principal component analysis (PCA) to assess the complete dataset of the samples. The PCA results showed that the samples were separated into two clusters. Correlations were calculated by the Pearson correlation coefficient and visualized in unclustered heatmaps. This demonstrated that the various groups had a distinct directionality based on similarities in gene expression (Figures 4A,B,C). We also identified the differentially expressed genes (DEGs). The volcano plots showed the DEG results from the treatment with DHA@Zif-8 NP. We used the Benjamini–Hochberg method to perform multiple testing corrections with a 1.5-fold change cutoff and a corrected *p*-value of <0.05. We compared the RNA-seq data from the C and D, and we verified 219 DEGs, among which 126 were downregulated and 123 were upregulated.

**Figure 5:**
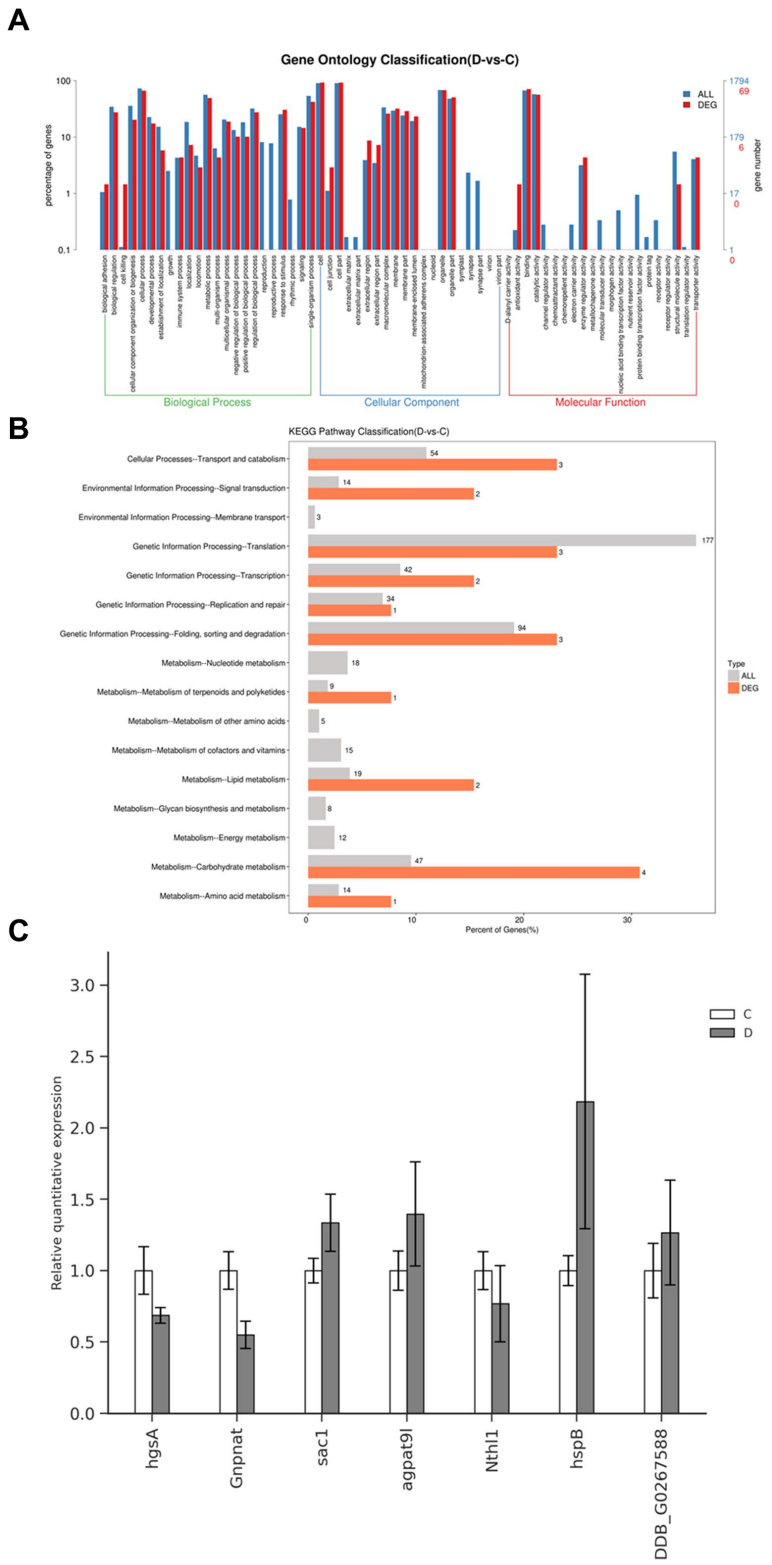
GO and pathway functional enrichment analysis of DEGs in DHA@Zif-8 NPs treated *G. lamblia*. **(A)** GO enrichment result. **(B)** Pathway functional enrichment of DEGs. **(C)** mRNA levels of four selected genes in *G. lamblia* as detected by qRT-PCR.

Using the RNA-seq data, we conducted pathway enrichment analysis and performed GO for 219 DEGs following treatment with DHA@Zif-8 NP. The GO classification covers three domains: biological processes (BPs), molecular functions (MF), and cellular components (CCs). A high proportion of DEGs in the BP domain was related to biological regulation, immune system processes, cellular processes, metabolic processes, and responses to stimuli. The enriched parts in the CC domain cover the cell, cell part, membrane, and organelle. The catalytic activity, binding, molecular function regulator, and transcriptional activator activity were affected primarily in the MF domain (Figure 5A). On the basis of the DEG results, we performed enrichment analysis and KEGG functional classification. According to the KEGG functional classification analysis, the treatment with DHA@Zif-8 NPs affected signal transduction as well as amino acid, carbohydrate, and lipid metabolism translation (Figure 5B). The KEGG pathway enrichment revealed that ribosome biogenesis in eukaryotes, phosphatidylinositol signaling system, and spliceosomes were affected (Table 1).

**Table 1:** KEGG pathway enrichment result.

Id	Term
gla00072	Synthesis and degradation of ketone bodies
gla00650	Butanoate metabolism
gla00561	Glycerolipid metabolism
gla00280	Valine, leucine and isoleucine degradation
gla00562	Inositol phosphate metabolism
gla04070	Phosphatidylinositol signaling system
gla03040	Spliceosome
gla04146	Peroxisome
gla04144	Endocytosis
gla03410	Base excision repair
gla00564	Glycerophospholipid metabolism
gla00900	Terpenoid backbone biosynthesis
gla03008	Ribosome biogenesis in eukaryotes
gla00520	Amino sugar and nucleotide sugar metabolism
gla04141	Protein processing in endoplasmic reticulum
gla03018	RNA degradation

Next, we selected some of the essential genes related to the metabolism to verify the expression levels in DHA@Zif-8 NP-treated or -untreated *G. lamblia* by quantitative reverse transcription polymerase chain reaction (qRT-PCR) (Figure 5C). According to the results of qRT-PCR, hgsA, Gnpnat, Nthl1, sac1, agpat9l, hspB, and DDB_G0267588 were downregulated. The RNA-seq results were the same as the gene expression.

### 2.4 Anti-*Giardia lamblia* effect *in vivo*

**Figure 6:**
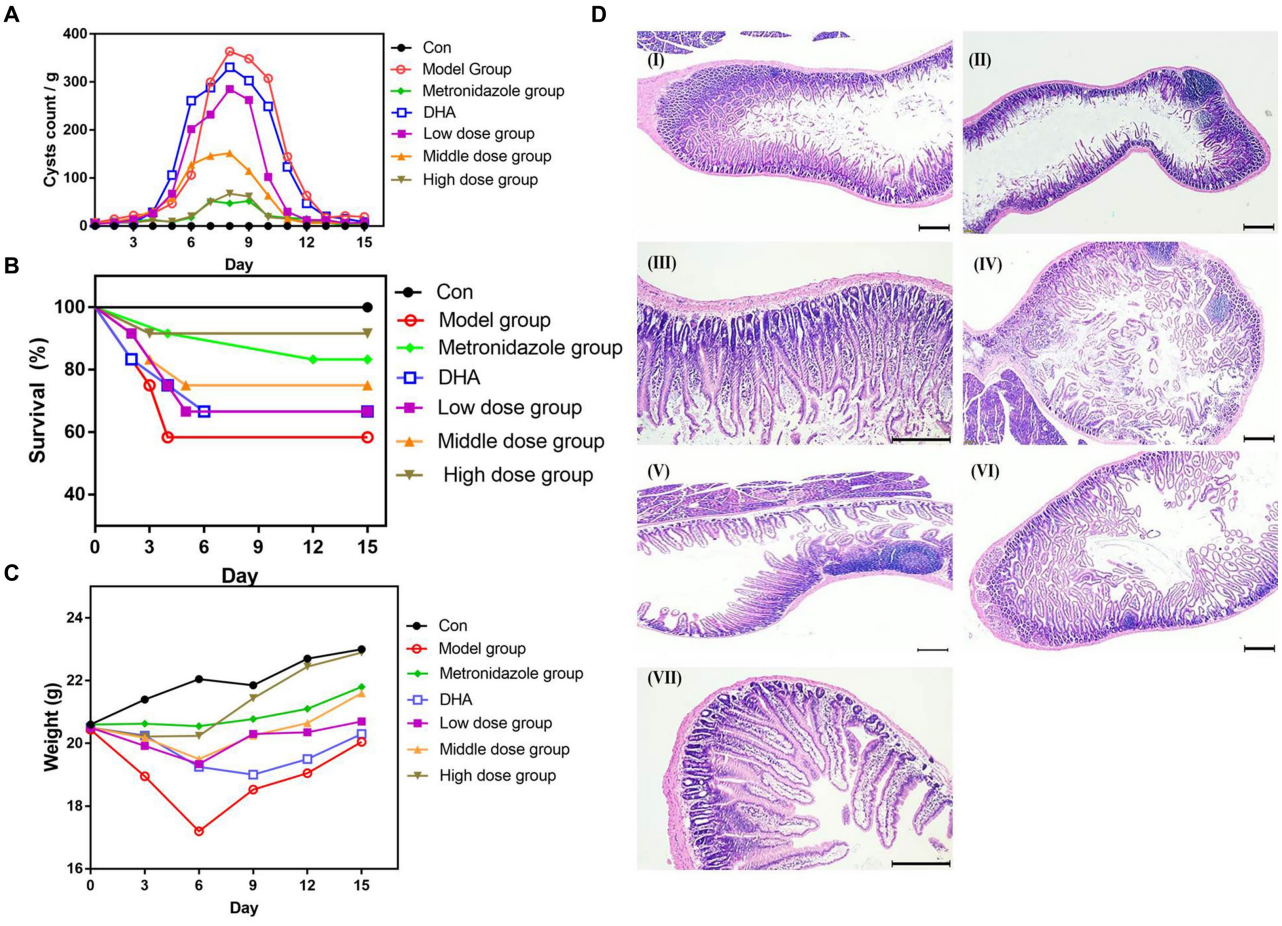
*In vivo* Anti-Giardial performance of DHA@Zif-8 NPs. **(A)** Fecal cyst counts in the stool were detected. **(B)** The survival rate of mice in each group. **(C)** The body weight profiles of mice during the experiment in each group. **(D)** H&E-stained images of small intestines collected from mice. **(I)** Control group; **(II)** *G. lamblia* infected group; **(III)** MTZ treated group; **(IV)** Free DHA treated group; **(V)** Low dose of DHA@Zif-8 NPs group; **(VI)** Middle dose of DHA@Zif-8 NPs group; **(VII)** High dose of DHA@Zif-8 NPs group. Scale bars: 200 μm.

We assessed the experimental infection by monitoring the weight of mice and fecal *Giardia* cysts. We found that mice had a slight decrease in weight, and *Giardia* cysts in the fecal matter were successfully confirmed as infection. Following DHA@Zif-8 NP treatment, we observed a significant decrease in the *G. lamblia* cyst count in the fecal samples from the infected treated mice relative to the infected nontreated mice. In addition, we did not observe any significant difference between high doses of DHA@Zif-8 NPs and MTZ. Following a high dose of DHA@Zif-8, the most obvious effect was the disappearance of the parasite from the stool and a more than 95% reduction after treatment for 12 days (Figure 6A). The DHA@Zif-8 NP-treated groups’ survival rate was significantly better than the infected groups (Figure 6B). After the experiment, the treatment group’s body weight was the same as the control group, whereas the model group’s body weight was lower (Figure 6C).

We conducted an H&E examination of sections of the small intestines in the mice in the control group. We observed normal brush border and villous architecture with an average length and width of the villi. In the infected group, we observed that the structure of the intestinal mucosa had changed as evident in atrophy and villous shortening. Along with fusion, this contributed to increased goblet cells, a blunting of most villi with desquamation, a decrease in the villous height–to–crypt length ratio, and infiltration of the lamina propria with inflammatory cells (e.g., lymphocytes and eosinophils) with diffuse loss of the brush border of the microvillous surface area and necrosis. In the DHA@Zif-8 NP-treated mice, we observed a marked improvement in histopathological lesions in the form of complete healing of the intestinal mucosa. In addition, mucosal ulceration was absent, villous architecture was normal, and the brush border was preserved. As shown in Figure 6D, inflammatory cell infiltration of the *G. lamina* disappeared, thus proving that DHA@Zif-8 NPs play a therapeutic role against giardiasis.

## 3 Discussion

DHA is an effective and fast-acting antimalarial drug with low toxicity. It is widely used for inhibiting pathogenic parasites, such as *G. lamblia*, *Toxoplasma gondii*, *Schistosoma japonicum*, *Neosporidium canis*, and *Leishmania* (20). According to previous research, by destroying cytoskeleton proteins and affecting the cell cycle, DHA inhibits *G. lamblia* proliferation (21, 22). However, the drug activity is not very satisfactory. It may be that the drug cannot be effective uptaked by *G. lamblia*. Encapsulating DHA in carrier materials or designing it as a polymer may significantly improve water solubility and bioavailability, which may provide new strategies to treat *G. lamblia*.

Zif-8, a well-studied MOF, has been investigated extensively in the literature for numerous applications, including microelectronics, catalysis, and drug delivery. Using an easy one-pot encapsulation strategy, Zif-8 can load drugs. This method varied from the postsynthesis strategy and the assembly strategy used by other MOFs (23). Because Zif-8 NPs have superior internal biodegradability, thermal stability, and pH-responsive properties, they stabilize under physiological conditions and decompose at low pH. *G. lamblia* is a facultative anaerobic eukaryote without mitochondria. It can use endogenously stored sugars and exogenous glucose to produce ethyl alcohol, acetic acid, and carbon dioxide under aerobic or anaerobic conditions (24, 25). For use in *G. lamblia*, a Zif-8 MOF-based low pH-sensitive drug delivery system is essential. In the current research, Zif-8 NP-encapsulated DHA attempted to address problems of poor water solubility and nonspecific delivery to enhance its antiprotozoal effects. DHA@Zif-8 NPs significantly improved the antigiardial effects of DHA and changed the ultrastructure’s swelling and the endoplasmic reticulum’s expansion. As we know, nanocarriers can improve the absorption and utilization of drugs, achieve efficient, targeted delivery, prolong the consumption half-life of drugs, and reduce harmful side effects on normal tissues (26). We used CLSM to detect whether *G. lamblia* exhibited the cellular uptake behavior of DHA@Zif-8 NPs. According to our research results, DHA@Zif-8 NPs featured an internalization mechanism that was time-dependent. This result verified that the endocytosis of DHA@Zif-8 NPs may have enhanced the inhibition effect.

RNA-seq has improved significantly over the past few decades and is not considered the most prominent approach used for transcriptome profiling (27). This method has clarified life activities at the molecular level and has supported research to reveal the transcriptome and genome. As complex organisms, parasites have a higher degree of biodiversity. A large number of parasites are being explored by human beings. It has been verified that insect species’ whole genome only accounts for a tiny part (28). Thanks to innovative high-throughput transcriptome sequencing technology, new instruments have been introduced to improve the quality and speed of sequencing. As a result, more accurate and timely technical support for whole-genome scanning of parasites is now available (29). We used RNA-seq in this study to detect any changes in gene expression. After DHA@Zif-8 NP treatment, 219 DEGs were identified, of which 123 were upregulated and 126 were downregulated. To determine the possible mechanisms by which module genes support the clinical features, GO and KEGG pathway enrichment are both essential. The GO and KEGG pathway enrichment results primarily affected the metabolic processes, such as amino acid, carbohydrate, and lipid metabolism.

It has been reported that antitumor and antimalarial mechanisms of DHA involve interfering with the function of mitochondria and affects energy metabolism (30). According to our previous studies, the energy metabolism of *Giardia* mainly depends on glycolysis (31). Under conditions such as sugar starvation, the energy metabolism pathway will convert glycolysis to amino acid metabolism. The formation of the *Giardia* cyst wall requires lipid metabolism. Therefore, energy is necessary for the growth and proliferation of *G. lamblia* (32). However, previous studies have not reported the effect of DHA on *Giardia* metabolism. Although it has been pointed out that DHA can destroy the cytoskeleton of *Giardia*, it is believed that the cytoskeleton component may be the target of artemisinin drugs. The limited drug intake in *Giardia* may cause this conclusion. Our research revealed that the anti-giardial mechanism of encapsulation DHA in Zif-8 inhibits metabolic processes.

*Giardia* has trophozoites and cysts, but the cysts and trophozoites alternate exist. The first stage is the cyst stage, which mainly lives in the external environment and looks for opportunities to infect the host (33). A prominent feature of the cyst is surrounded by a very thick cell wall, which has a specific resistance to adverse external influences. In the host, the formation of cysts under the influence of drugs (metronidazole) and immune response are the main factors responsible for Giardiasis’ chronicity and drug resistance (34). Common pesticides do not affect Giardia cysts, which is also an important factor in spreading Giardiasis worldwide (35). Therefore, it is essential to discover a drug that can kill the trophozoites, inhibit cyst formation from curing Giardiasis, and cut off its transmission. Our results showed that DHA@Zif-8 NPs could inhibit *G. lamblia* encystation. *Giardia* has a highly developed cytoskeleton system consisting of microtubules, microfilaments, and cytoskeleton proteins. Cytoskeleton proteins of *Giardia* are closely related to its pathogenicity. The α-tubulin and β-tubulin could form heterodimers, the two main types of tubulin-forming microtubules (36). Microfilaments in the cytoskeleton related to encystation are formed by actin, which is part of the globular multifunctional protein family (37). Cwp2 is an important cyst wall protein of *Giardia*, which is essential in *Giardia* cyst formation (38). In the DHA@Zif-8 NP-treated group, the expression levels of Cwp2 were lower and the expression levels of actin, α-tubulin, and β-tubulin were higher. The certified DHA@Zif-8 inhibited cyst formation. Accordingly, our drug offered more advantages than metronidazole in antigiardial mechanisms.

The resistance of *G. lamblia* to routine therapy has become an increasingly serious problem. To address this problem, novel, alternative therapeutic agents that may work against *G. lamblia* and that have only slight side effects are being explored. We assessed whether DHA@Zif-8 NPs had a therapeutic effect on *G. lamblia in vivo*. According to our results, the number of intestinal trophozoites and fecal *G. lamblia* cysts was significantly lower than in the infected groups. Our histopathological results demonstrated that the effect on intestinal wall histopathology can be enhanced more by DHA@Zif-8 than by MTZ and DHA. We attributed this to the mechanisms of DHA@Zif-8’s improved drug delivery, which produced high amounts of ROS and inhibited cyst formation.

## 4 Conclusion

In this study, we prepared DHA-loaded Zif-8 NPs that were water-dispersible. We conducted *in vitro* experiments and found that DHA@Zif-8 NPs improved the antigiardial effect better than free DHA because cyst formation was inhibited and DHA uptake was enhanced. The RNA-seq analysis results showed that the most likely antigiardial mechanism of DHA@Zif-8 NPs was the inhibition of metabolic processes. In addition, we considered DHA@Zif-8 NPs to be a pioneering therapy in eliminating *Giardia* infection *in vivo*. The clinical application and development of DHA@Zif-8 for protists will rely on these findings.

## 5 Materials and methods

### 5.1 Materials and equipment

We purchased the chemicals and solvents from commercial sources. Unless indicated otherwise, all were used without further treatment. Molecular Devices supplied the SpectraMax iD5 Multi-Mode Microplate Reader. Becton, Dickinson, and Company (BD) (Franklin Lakes, NJ, USA) supplied the Accuri C6 flow cytometer. We used a JEOL JEM-1011 electron microscope (acceleration voltage of 100 kV) for the TEM images.

### 5.2 One-pot synthesis of DHA@Zif-8

We followed the method given by Li (39) to perform the synthesis of DHA@Zif-8. We dissolved 75 mg of zinc nitrate hexahydrate in 2.5 mL of deionized water in a typical experiment. In other cases, we dissolved 165 mg of 2-methyl imidazole in 4.5 mL of methanol and 10 mg of DHA in 0.5 mL of DMF. We added the aqueous zinc nitrate solution to the 2-methyl imidazole and DHA solution under continuous stirring at room temperature. As a result, the synthesis solution turned turbid quickly. The DHA@Zif-8 NPs formed after 3 min. To obtain DHA@Zif-8 NPs, we centrifuged the solution at 12,000 rpm for 10 min. To remove the unreacted reagents, we washed the DHA@Zif-8 NPs with methanol three times. We collected the supernatants to measure encapsulation efficiency and drug loading content of DHA. Following treatment with 0.2% NaOH aqueous solution at 50°C for 30 min converted DHA in the supernatant into a UV-absorbing compound. We used the UV–vis spectrometer to measure the detection wavelength at 290 nm. After freeze-drying, we stored the products at −20°C until further use. We used the method to synthesize the Zif-8 NPs as a control.

### 5.3 Cultivation of *Giardia lamblia*

We collected C2, trophozoite of *G. lamblia* isolates, from a patient in southwest China. We used modified trypticase yeast extract iron-serum-33 medium (TYI-S-33), which was composed of 2% casein digest, 1% yeast extract, 1% glucose, 0.2% NaCl, 0.2% l-cysteine, 0.02% ascorbic acid, 0.2% K_2_HPO_4_, and 0.06% KH_2_PO_4_. We used borosilicate glass screw-cap culture tubes to supplement this with 10% heat-inactivated bovine serum (Hangzhou Sijiqing Biological Engineering Materials Company) and 0.05% bovine bile (Sigma) at pH 6.8. Without shaking, we inoculated the culture with 4 × 10^3^ trophozoites per 4 mL tube at 37°C three times a week.

### 5.4 Effects of DHA@Zif-8 NPs on *Giardia lamblia* growth

In the TYI-S-33 medium, pH 6.8, we grew Trophozoites of *G. lamblia* at 37°C for 48 h. This medium was supplemented with 0.05% bovine bile and 10% heat-inactivated bovine serum. We detached the trophozoites in the logarithmic phase by chilling. To detect growth inhibition of DHA@Zif-8 NPs on *G. lamblia*, we subcultured the *G. lamblia* (5 × 10^4^) trophozoites with TYI-S-33 medium at 37°C for 24, 48, and 72 h in a 4 mL tube in the presence of various concentrations of DHA@Zif-8, DHA, or pure Zif-8 NPs. We counted the parasites every 24-h period for consecutive days. To make each reading, we placed a tube of cultured *G. lamblia* on ice for 20 min. Then, we inverted the tube gently to detach the trophozoites. We centrifuged the tubes for 10 min at 2,000 g. After we discarded the supernatants, we resuspended the pellets in 1 mL of PBS. Following homogenization, we took out the aliquots to count the parasites in the Neubauer chamber. We performed three experiments independently.

### 5.5 Effects of DHA@Zif-8 NPs on *Giardia lamblia* encystation

We grew *G. lamblia* for 48 h at 37°C and used chilling PBS to detach the trophozoites. We subcultured the trophozoites (5 × 10^4^) in a 4 mL tube with encystation medium TYI-S-33 (pH 7.1), which was supplemented with 0.5 mg/mL bile. After the *G. lamblia* reached the logarithmic growth period, we changed the medium to TYI-S-33 (pH 7.8) and added 10 mg/mL bile for 24 h. We next changed the medium to TYI- and added S-33 (pH 7.1) with 0.5 mg/mL bile for 24 h. We separated the *G. lamblia* into DHA@Zif-8 NP-treated, metronidazole-treated, and encystation groups (100 μg/mL). We used deionized water to split trophozoites and incomplete cysts, added 1 mL of distilled water, and used the blood cell counting plate to calculate the rate of cyst formation. After we extracted the total RNA, we reverse-transcribed it into single-stranded DNA. We used qPCR to detect the gene expression level of actin, *Cwp2*, α-tubulin, β-tubulin, HSP70, and GAPDH (see Supplementary Table S1 for the primer sequences).

### 5.6 Ultrastructural changes with TEM

We treated trophozoites for 48 h with various concentrations of DHA@Zif-8 NPs. Following DHA@Zif-8 NP treatment, we collected the trophozoites and fixed the sample in PBS (pH 7.3) with ice-cold 2.5% glutaraldehyde. We removed the fixative and washed each trophozoite two times with the cold PBS. Then, we used graded ethanol for dehydration, propylene oxide for immersion, and epoxy resin to embed the sample. We used lead citrate/uranyl acetate to double-stain ultra-thin sections, which we then examined by TEM.

### 5.7 ROS detection

We used a ROS assay kit (Beyotime, Shanghai, China) to assess the accumulation of intracellular ROS after DHA@Zif-8 NP treatment. We treated the trophozoites with various concentrations of DHA@Zif-8 NPs before incubation with 10 μM DCFH-DA for 30 min at 37°C. Then, we collected the samples and suspended them in ice-cold PBS. After deacetylation, DCFH-DA was oxidized by ROS and formed 2,7-dichlorofluorescein (DCF). We used flow cytometry at an excitation wavelength of 488 nm and an emission wavelength of 525 nm to measure the DCF fluorescence intensities.

### 5.8 *Giardia lamblia* uptake of DHA@Zif-8 NPs

We examined the DHA@Zif-8 NPs internalized by *G. lamblia* using a fluorescence microscope. We seeded the trophozoites in six-well culture plates at a density of 5 × 10^4^ cells per well. We put a sterile coverslip in each well and allowed them to adhere for 24 h. We treated the trophozoites with DHA@Zif-8 NPs at 37°C for 0.5, 2, and 4 h. We carefully removed the supernatant and washed the trophozoites three times with PBS. We fixed the cells with 500 μL of 4% formaldehyde at room temperature for 20 min and used PBS to wash them two times. We used a blue channel for Hoechst 33258 and a red channel for NR to visualize the trophozoites with a fluorescence microscope.

### 5.9 RNA-seq analysis

To conduct the RNA-seq analysis, we used trophozoites treated with DHA@Zif-8 NPs and performed the analysis for the DHA@Zif-8 NP-treated or untreated groups on three biological replicates. After this treatment, we used a mirVana miRNA Isolation Kit (Ambion, Austin, TX, USA) to extract RNA using the manufacturer’s recommendations. We treated the sample with RNase-free DNase I (ThermoFisher Scientific, Waltham, MA, USA) and removed the contaminated DNA. We used a NanoDrop 2000 spectrophotometer (Nanodrop Technologies, Wilmington, DE, USA) to assess the extracted RNA quality.

We used the RNA Nano6000 Assay Kit on a Bioanalyzer 2,100 system (Agilent Technologies, Santa Clara, CA, USA) to detect RNA integrity. If the samples had an RNA Integrity Number (RIN) of more than seven, we used them to construct the sequencing libraries. We constructed the libraries with a TruSeq Stranded mRNA LTSample Prep Kit (Illumina, San Diego, CA, USA) following the manufacturer’s instructions. We used the Illumina sequencing platform (HiSeq™ 2,500) to sequence the libraries and generated 150-bp paired-end reads. We filtered the raw tag data. We removed reads containing adapters, poly-N, and low-quality reads from the raw data to obtain clean data. On the basis of the high-quality clean data, we conducted the downstream analyses.

We performed DEG analyses to compare the DHA@Zif-8 NP-treated and untreated groups. First, we mapped clean reads to the reference genome using hisat2. Then, we calculated the FPKM and read count values for each transcript (protein coding) with bowtie2. Next, we identified the DEGs using the DESeq functions estimateSizeFactors and nbinomTest. For significant differential expression, we set a fold change of >1.5 or < 0.5 and a *p*-value of <0.05 as the threshold. Finally, to explore the transcript expression patterns, we performed a hierarchical cluster analysis of the DEGs. We used R based on the hypergeometric distribution to perform GO enrichment and KEGG pathway enrichment analysis.

### 5.10 qRT-PCR analysis

We used total RNA that was extracted from DHA@Zif-8 NP-treated and untreated trophozoites to characterize the expression of selected genes. We employed oligonucleotide primers that were gene-specific to perform qRT-PCR on RNA samples (Supplementary Table S1). We also used an iScript One-Step RT-PCR Kit with SYBR Green and CFX96 Touch Real-Time PCR Detection System (Bio-Rad, Hercules, CA, USA). All values were standardized to the control condition and normalized to β-actin. The standard error of the mean (SEM) was represented by error bars. We used Student’s *t*-test to assess statistical significance. After RT-PCR was complete, we obtained the C_t_ values from the ABI 7500 fast v2.0.1 software. To represent mRNA-fold change, we used the ^ΔΔ^Ct method.

### 5.11 Treatment efficiency *in vivo*

The Institutional Animal Care and Use Committee at Ji Lin Medical University approved all animal procedures (2024-LW001). The Changchun Yisi Laboratory Animal Technology Co., Ltd., provided the BALB/c mice, which we housed at a controlled room temperature and humidity with a 12 h photoperiod. Mice were provided with sterilized food and water. After 48–72 h, we harvested the *G. lamblia* trophozoites in TYI-S-33 medium in the log phase. One time every day, we used oral gavage to infect 50 of the mice with 1 × 10^6^ *G. lamblia* trophoblast and 1 × 10^5^ *G. lamblia* cyst in 0.2 mL of PBS. *Giardia* infection was monitored through daily fecal collection and cyst detection. We confirmed that the model was established successfully when the mice had higher *Giardia* colonization with the fecal *Giardia* cyst. We randomly divided the mice into seven groups. Each group had six mice: *Giardia* infection, metronidazole (10 μM /kg/d), free DHA (20 μM/kg/d), low dose (5 μM/kg/d), middle dose (10 μM/kg/d), and high dose of DHA@Zif-8 (20 μM/kg/d), and control. Mice was administered by intragastric administration once everyday. We collected fecal samples every day. After 15 days of observation, we killed the mice by cervical dislocation.

### 5.12 Histopathological examination

We removed the small intestine from each mouse aseptically. After we fixed the sample in 4% paraformaldehyde solution, it was embedded in paraffin. To monitor the kinetics of infection, we sliced and stained the sample with hematoxylin and eosin (H&E).

### 5.13 Statistical analysis

We performed all experiments at least three times. We expressed all results as the mean ± standard deviation. To determine the statistical difference between experimental and control groups, we used Student’s *t*-test. Significance is denoted by *p < 0.05.

## Data availability statement

All sequencing data are available through the NCBI Sequence Read Archive under the accession number PRJNA1102394. The raw reads of our transcriptome data have been deposited into the NCBI Short Read Archive (SRA, http://www.ncbi.nlm.nih.gov/sra/) under accession number SRR28745611-SRR28745616.

## Ethics statement

The animal study was approved by Approved file of Ethical Committee Jilin Medical University. The study was conducted in accordance with the local legislation and institutional requirements.

## Author contributions

XJ: Methodology, Validation, Writing – original draft, Writing – review & editing. YL: Methodology, Validation, Writing – original draft. SL: Data curation, Writing – original draft, Writing – review & editing. HS: Investigation, Writing – original draft. MZ: Investigation, Methodology, Writing – original draft. XW: Methodology, Validation, Writing – original draft. WZ: Writing – original draft, Writing – review & editing. XF: Funding acquisition, Project administration, Visualization, Writing – review & editing.
